# Association of post-COVID phenotypic manifestations with new-onset psychiatric disease

**DOI:** 10.1038/s41398-024-02967-z

**Published:** 2024-06-08

**Authors:** Ben Coleman, Elena Casiraghi, Tiffany J. Callahan, Hannah Blau, Lauren E. Chan, Bryan Laraway, Kevin B. Clark, Yochai Re’em, Ken R. Gersing, Kenneth J. Wilkins, Nomi L. Harris, Giorgio Valentini, Melissa A. Haendel, Justin T. Reese, Peter N. Robinson

**Affiliations:** 1 The Jackson Laboratory for Genomic Medicine, Farmington, CT USA; 2 Institute for Systems Genomics, University of Connecticut, Farmington, CT USA; 3 AnacletoLab, Dipartimento di Informatica, Università degli Studi di Milano, Milan, Italy; 4 Division of Environmental Genomics and Systems Biology, Lawrence Berkeley National Laboratory, Berkeley, CA USA; 5 Department of Biomedical Informatics, Columbia University Irving Medical Center, New York, NY USA; 6 Department of Pediatrics, University of Chicago, Chicago, IL USA; 7 University of North Carolina at Chapel Hill, Chapel Hill, NC USA; 8 Cures Within Reach, Chicago, IL USA; 9 Champions Service, Computational Science Support Network, Multi-Tier Assistance, Training, and Computational Help (MATCH) Program, National Science Foundation Advanced Cyberinfrastructure Coordination Ecosystem: Services and Support (ACCESS),; 10 Neurology Subgroup, COVID-19 International Research Team, https://www.cov-irt.org/; 11 Weill Cornell Medicine, Department of Psychiatry, New York, NY USA; 12 National Center for Advancing Translational Sciences, National Institutes of Health, Bethesda, MD USA; 13 Biostatistics Program, National Institute of Diabetes and Digestive and Kidney Diseases, National Institutes of Health, Bethesda, MD USA

**Keywords:** Depression, Psychiatric disorders

## Abstract

Acute COVID-19 infection can be followed by diverse clinical manifestations referred to as Post Acute Sequelae of SARS-CoV2 Infection (PASC). Studies have shown an increased risk of being diagnosed with new-onset psychiatric disease following a diagnosis of acute COVID-19. However, it was unclear whether non-psychiatric PASC-associated manifestations (PASC-AMs) are associated with an increased risk of new-onset psychiatric disease following COVID-19. A retrospective electronic health record (EHR) cohort study of 2,391,006 individuals with acute COVID-19 was performed to evaluate whether non-psychiatric PASC-AMs are associated with new-onset psychiatric disease. Data were obtained from the National COVID Cohort Collaborative (N3C), which has EHR data from 76 clinical organizations. EHR codes were mapped to 151 non-psychiatric PASC-AMs recorded 28–120 days following SARS-CoV-2 diagnosis and before diagnosis of new-onset psychiatric disease. Association of newly diagnosed psychiatric disease with age, sex, race, pre-existing comorbidities, and PASC-AMs in seven categories was assessed by logistic regression. There were significant associations between a diagnosis of any psychiatric disease and five categories of PASC-AMs with odds ratios highest for neurological, cardiovascular, and constitutional PASC-AMs with odds ratios of 1.31, 1.29, and 1.23 respectively. Secondary analysis revealed that the proportions of 50 individual clinical features significantly differed between patients diagnosed with different psychiatric diseases. Our study provides evidence for association between non-psychiatric PASC-AMs and the incidence of newly diagnosed psychiatric disease. Significant associations were found for features related to multiple organ systems. This information could prove useful in understanding risk stratification for new-onset psychiatric disease following COVID-19. Prospective studies are needed to corroborate these findings.

## Introduction

Severe acute respiratory syndrome coronavirus 2 (SARS-CoV-2), the virus that causes coronavirus disease 2019 (COVID-19), is responsible for over 650 million cases with 6 million deaths worldwide [1]. Many patients experience manifestations that persist following acute COVID-19 or with onset after the acute period, affecting various organ systems [2, 3]. These manifestations, when not explained by another cause, are part of the broader syndrome of Post Acute Sequelae of SARS-CoV2 Infection (PASC; colloquially referred to as Long COVID). The rate of newly diagnosed psychiatric disease has been found to be significantly increased in patients following COVID-19 infection. The most significant risk has been demonstrated for anxiety disorders, with hazard ratios ranging from 1.3-2.1 [4–8]. In a prior study, we found that this risk was only significant in early post-acute phase (28-120 days following COVID-19 diagnosis), reflecting a timeframe when these psychiatric sequelae are most likely to be diagnosed [5]. A smaller study using electronic health record (EHR) data from a Japanese cohort similarly showed that COVID-19 patients were more likely to receive a diagnosis of psychiatric disease one to three months after COVID-19 compared to controls with influenza or respiratory tract infections [9]. These findings have major public health ramifications, creating a need to further characterize the risk of newly diagnosed psychiatric disease following COVID-19.

One relevant question is how psychiatric sequelae relate to other manifestations of PASC. Acute COVID-19 may be characterized by neurological manifestations such as confusion, stroke, and neuromuscular disorders. Pathomechanisms include viral neuroinvasion, immune activation and inflammation within the central nervous system (CNS), and endotheliopathy associated with blood–brain barrier dysfunction, which could additionally lead to psychiatric manifestations through diverse pathophysiological mechanisms [10]. Following acute COVID-19, some patients exhibit manifestations including fatigue, headache, difficulty concentrating, cognitive impairment, anxiety and mood disorders, and dysautonomia [11]. The pathobiology of these post-COVID manifestations are thought to be the result of a combination of delayed recovery from inflammation, viral persistence, and autoimmunity resulting from infection [12]. The relationship between newly diagnosed psychiatric sequelae and other PASC manifestations has not been well characterized. One challenge in understanding the pathogenesis of the psychiatric manifestations of PASC is the fact that it is not a single, well-delineated disease; instead, PASC has multiple distinct pathogenetic mechanisms that may underlie different psychiatric manifestations [13].

In this study, we performed a detailed analysis of 151 PASC-associated manifestations (PASC-AMs) encoded using terms of the Human Phenotype Ontology (HPO), which is widely used to support differential diagnosis and translational research in human genetics [14]. We analyzed 2,391,006 patients for whom at least 120 days of follow-up data were available after acute COVID-19 in multicenter EHR data from the National COVID Cohort Collaborative (N3C) [15]. We show significant associations between new-onset psychiatric disease and PASC-AMs from five organ systems.

## Methods

### Study population and data sources

In this retrospective cohort study, we examined the association between PASC-AMs and diagnosis of new-onset psychiatric disease and four subcategories: anxiety disorder, depressive disorder, psychosis, and substance abuse disorder following acute COVID-19. We used patient data accessed through the N3C Data Enclave [15]. N3C has integrated EHRs from 76 clinical organizations in the United States. We analyzed N3C data frozen on November 3, 2023, which comprised records for 8 million COVID-19 positive patients. Data included over 30 billion rows of data and 21 million patients. Data were collected from the clinical organizations, normalized to the Observational Medical Outcomes Partnership (OMOP) 5.3.1 vocabulary [16], then de-identified and made available to participating N3C research institutions. The study was exempted by the Institutional Review Board (IRB) at the Jackson Laboratory under 45 CFR 46.101(b) (Common Rule). The N3C data transfer to the National Center for Advancing Translational Sciences (NCATS) is performed under a Johns Hopkins University Reliance Protocol # IRB00249128 or individual site agreements with NIH. The N3C Data Enclave is managed under the authority of the NIH; information can be found at https://ncats.nih.gov/n3c/resources.

### Exposures and outcomes

Clinical data including comorbidities, medications, and outcomes were identified using concept identifiers in the OMOP common data model. Demographics, laboratory values, COVID-19 status, and psychiatric diagnoses were collected for each patient.

Patients were included in the primary analysis if SARS-CoV-2 was detected by polymerase chain reaction (PCR) or antigen test after January 1, 2020. Patients with a history of any psychiatric disease prior to 28 days after COVID-19 diagnosis and patients without a medical record covering at least a year prior to and 120 days after COVID-19 diagnosis were excluded from this analysis (Supplemental Fig. S1).

**Fig. 1:**
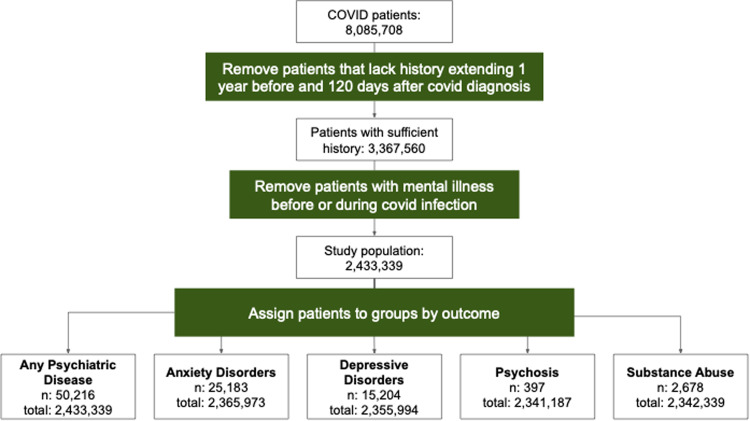
workflow for creating the cohort. We selected all patients with a positive PCR or antigen test for COVID-19 who had records extending greater than 1 year prior to COVID-19 infection and at least 120 days after diagnosis of COVID-19. Patients with any record of psychiatric disease prior to the post-COVID phase (28 days after diagnosis) were removed. For each outcome, patients with more than one outcome (anxiety disorder, depressive disorder, psychosis, and substance abuse) whose first psychiatric diagnosis was not the outcome of interest were excluded from the analysis. We report the number of patients included with the outcome (n) and the total number of patients.

We identified patients with a diagnosis of any psychiatric disease and subcategories of anxiety disorder, depressive disorder, psychosis, and substance abuse using diagnostic codes defined by OMOP. We treated each category of psychiatric disease as an outcome. Psychiatric outcomes were considered if they were first diagnosed in the period of 28 to 120 days following COVID-19 diagnosis (Fig. 1, Supplemental Fig. S1).

We studied known PASC-AMs that had been previously described using 287 Human Phenotype Ontology (HPO) terms [2, 17]. We mapped 176 of these terms to terms in the OMOP data model using the OMOP2OBO mapping algorithm, which leverages well-established automatic mapping techniques and manually-derived expert-verified annotations to align concepts from each source (some HPO terms mapped to multiple OMOP terms and some HPO terms had no good OMOP counterparts) [13, 18]. We removed 15 HPO terms from our list of non-psychiatric PASC-AMS because they described psychiatric phenotypes, leaving 161 non-psychiatric HPO terms. 151 of these terms were found in the EHR data and were used for the final analysis. In this way, we were able to identify when patients experienced HPO-defined PASC-AMs using OMOP-encoded patient records. We then categorized HPO terms by affected system. These categories included cardiovascular, constitutional, endocrine, ear-nose-throat (ENT), eye, gastrointestinal, immunology, laboratory, neurological, pulmonary, and skin (Supplemental Table S2). The HPO terms were recorded in the time period from 28 days after onset of acute COVID-19 up to either the occurrence of newly diagnosed psychiatric disease or 120 days (if no diagnosis of psychiatric disease was recorded) (Supplemental Fig. S1).

In addition to PASC-AMs, we considered the effects of patient demographics, including age, race and ethnicity, sex, smoking status, BMI, visit type (inpatient or outpatient), length of stay (if applicable). We also considered pre-COVID comorbidities (Table 1). All comorbidities were defined as a binary variable indicating whether that patient had received the diagnosis of the comorbidity prior to COVID diagnosis. Only comorbidities present in more than 1% of the population were included in the final analysis to avoid biased estimates (Table 1) [19].

**Table 1:** Characteristics of the study cohort are presented as counts (with percentage for categorical/Boolean variables) or as mean ± standard deviation (for numeric variables).

Psychiatric Diagnosis	Number of patients	Percentage of patients
Any Psychiatric Disease	50216	2.10%
Anxiety Disorder	25183	1.05%
Depressive Disorder	15204	0.64%
Psychosis	397	0.02%
Substance Abuse	2678	0.11%
None	2375802	99.36%

HPO categories	Number of patients	Percentage of patients
Any PASC-AM	305360	12.77%
constitutional	98833	4.13%
gastrointestinal	87310	3.65%
neurological	86663	3.62%
pulmonary	71247	2.98%
ENT	39501	1.65%
cardiovascular	37434	1.57%
endocrine	36021	1.51%
laboratory	18130	0.76%
eye	11984	0.50%
skin	11591	0.48%
immunology	2236	0.09%

Race, Ethnicity	Number of patients	Percentage of patients
White, Non-Hispanic	1552583	64.93%
Black or African American, Non-Hispanic	317162	13.26%
Hispanic or Latino, Any Race	290577	12.15%
Unknown	113374	4.74%
Asian, Non-Hispanic	64212	2.69%
Other, Non-Hispanic	38760	1.62%
Native Hawaiian or Other Pacific Islander, Non-Hispanic	5515	0.23%

BMI (30.3 ± 9.1)	Number of patients	Percentage of patients
BMI < 20	108123	11.32%
20 ≤ BMI < 25	168792	17.67%
25 ≤ BMI < 30	245372	25.68%
30 ≤ BMI < 35	203517	21.30%
35 ≤ BMI < 40	117392	12.29%
BMI ≥ 40	112308	11.75%

Hospital stay	Number of patients	Percentage of patients
Outpatients (Length of stay = 0 days)	2299720	96.18%
Inpatients (6.1 ± 9.7)	91286	3.82%
Length of stay = 1 days	7071	0.30%
Length of stay = 2, 3 days	28698	1.20%
Length of stay = [4–7] days	32823	1.37%
Length of stay > 7 days	22694	0.95%

Comorbidities	Number of patients	Percentage of patients
hypertension	439125	18.37%
neoplasm	320691	13.41%
non-ischemic heart disease	242985	10.16%
chronic respiratory disease	204982	8.57%
type 2 diabetes	189753	7.94%
non-hypertensive chronic kidney disease	82864	3.47%
nicotine_dependence	75285	3.15%
ischemic_heart_disease	56842	2.38%
other_liver_disease	39212	1.64%
hepatic_steatosis	37486	1.57%
hypertensive kidney disease	36074	1.51%
cerebral_infarction	18383	0.77%
malignant lymph/blood cancer	17887	0.75%
psoriasis	16788	0.70%
rheumatoid_arthritis	16692	0.70%
type 1 diabetes	13731	0.57%
hepatic_fibrosis	9021	0.38%
lupus	6151	0.26%
chronic_hepatitis	5126	0.21%
portal_hypertension	2759	0.12%
hepatic_failure	2122	0.09%
alcoholic_liver_damage	1157	0.05%
hepatic passive congestion	509	0.02%
immunosuppression	380	0.02%

Sex	Number of patients	Percentage of patients
FEMALE	1336507	55.90%
MALE	1053580	44.06%
Other or Unknown	919	0.04%

Smoking status	Number of patients	Percentage of patients
Non-smoker	1120072	98.60%
Current or Former	15901	1.40%

Age (40.5 ± 21.2)	Number of patients	Percentage of patients
age < 10	322093	13.47%
10 ≤ age ≤ 19	260236	10.88%
20 ≤ age ≤ 29	328781	13.75%
30 ≤ age ≤ 39	338379	14.15%
40 ≤ age ≤ 49	321769	13.46%
50 ≤ age ≤ 59	331475	13.86%
60 ≤ age ≤ 69	271395	11.35%
70 ≤ age ≤ 79	163010	6.82%
age ≥ 80	53868	2.25%

The percentage is calculated with respect to the size of the entire group (2,391,006). Comorbidities and HPO categories only present in less than 1% of the cohort were removed from the analysis.

### Statistical analysis

Data analysis was performed using Palantir Foundry (Palantir Technologies Inc., Denver, Colorado). The analysis was structured as a directed acyclic graph of data transformations. Individual transformations were implemented as nodes consisting of SQL, Python (version 3.6.10), or R (version 3.5.1) code.

To address data missingness, we applied a multiple imputation strategy and computed pooled estimates by applying Rubin’s rule [20]. BMI was the most commonly missing variable (in 58% of cases). We imputed BMI six times with the missRanger algorithm and applied a multiple imputation estimation pipeline to derive the log odds estimates. The missRanger algorithm was chosen based on a previous study comparing different multiple imputation techniques on an N3C cohort of diabetic patients [21]. We identified all HPO-defined PASC-AMs that occurred between 28 and 120 days following COVID-19 diagnosis. PASC-AMs that were first documented after or on the same day as the diagnosis of a psychiatric disease were not included in the analysis. For each patient, we counted the number of unique HPO terms in each of the eleven categories described in the preceding section. PASC-AM categories present in less than 1% of the population were not considered.

To investigate the association of PASC manifestations and other covariates with newly diagnosed psychiatric disease, we performed logistic regression using the *glm* function in R. The predictors included age, sex, race, pre-existing comorbidities, and the counts of manifestations in each of the eleven HPO categories. Only predictors present in more than 1% of the cohort were used. We conducted separate analyses to predict the occurrence of psychiatric disease and four subcategories: anxiety disorder, depressive disorder, psychosis, substance abuse, ADHD, and bipolar disorder. The control group was patients with no psychiatric diagnosis. For each regression, we recorded the estimated odds ratio, 95% confidence intervals, and corresponding p-value.

To better understand the temporal relationship between PASC-AMs and psychiatric diseases, we looked at the distribution of patients by time between PASC-AM and outcome. Non-exclusive groups were created for each PASC-AM category that contained any patient with the respective PASC-AM and any psychiatric outcome. Histograms were created to show the distribution across time (ranging 1-92 days) for patients in each group.

We performed a chi-squared test of independence to assess if the incidence of HPO terms in each category were significantly associated with an outcome. For each term, a contingency table was constructed containing the counts of study participants with or without the corresponding HPO annotation and with or without the corresponding new-onset psychiatric disease. For the chi-squared analysis, we removed 6,208 patients (8.1% of those with any psychiatric diagnosis) who had more than one psychiatric outcome. P-values were adjusted using Bonferroni correction.

### Sensitivity analyses

PASC-AMs were only considered if they occurred prior to a diagnosis of psychiatric outcome or the 120-day cutoff. As a result, PASC-AMs were recorded over a longer period for patients without a diagnosis of a psychiatric disease. This leads to more conservative results with odds ratios that may be negatively shifted. To assess this effect, we computed the average time of receiving a psychiatric diagnosis for patients in the outcome group (72 days) and then repeated the logistic regression after excluding PASC-AMs in the control group that occurred after this time point.

Imputation was used to compensate for substantial missingness of BMI data. Since BMI is associated with risk of severe COVID-19 [22] and could therefore act as a confounder, we also performed the analysis without imputation, keeping only patients with complete data. To ascertain whether our results are consistent between inpatients and outpatients, we separated our cohorts into those who were admitted for their COVID-19 infection and those who were not.

Previous studies that investigated PASC and related manifestations have used different criteria to identify whether a manifestation is attributed to acute COVID-19 or PASC. To determine whether this had a large effect on our results, we repeated our regression analysis using the alternative definition of 42 days after initial diagnosis for outpatients or discharge for inpatients to 365 days after initial diagnosis.

In this study, we investigate the association between PASC-AMs and subsequent psychiatric disease. In our main analysis, we assume that psychiatric diseases diagnosed after PASC-AMs likewise have an onset subsequent to the date on which PASC-AMs were recorded. We performed an additional sensitivity analysis to assess whether our results could have been impacted by a greater delay between onset of psychiatric disease and diagnosis and recording of the diagnosis in the EHR. For this, we excluded PASC-AMs that were diagnosed within ten days prior to the psychiatric diagnosis, and otherwise repeated the analysis unchanged.

### Role of the Funding source

The funders had no role in study design, data collection, analysis, interpretation, manuscript writing, or the decision to submit for publication. The corresponding authors had full access to all study data and had final responsibility for the decision to submit for publication.

## Results

A total of 8 million patients with prior COVID-19 were assessed. We restricted analysis to patients with no previous recorded psychiatric disease and at least one year of data prior to acute COVID-19 diagnosis and 120 days after. This left 2,391,006 patients in the COVID-19 cohort. We found that 2.1% of patients had a newly diagnosed psychiatric disease in the early post-acute phase (28-120 days after COVID-19 diagnosis) (Table 1).

The outcomes of interest were psychiatric diagnoses and four subcategories: anxiety disorder, depressive disorder, psychosis, and substance abuse. To identify risk factors we exploited the hierarchical structure of the HPO to group into eleven categories the HPO terms describing clinical manifestations that may be observed in PASC [2]. We refer to these manifestations as PASC-AMs to reflect the difficulty of inferring causal relationships on the basis of EHR data. The patient’s symptomatology in each category is summarized by an integer-valued variable equal to the number of distinct HPO terms recorded in that category. Constitutional PASC-AMs were most commonly reported, being found in 4.13% of patients. A logistic regression was performed with these variables as well as binary variables derived from 25 pre-existing comorbidities and age, sex, and race. We then removed categories and variables that were recorded in less than 1% of the cohort (Table 1).

**Fig. 2:**
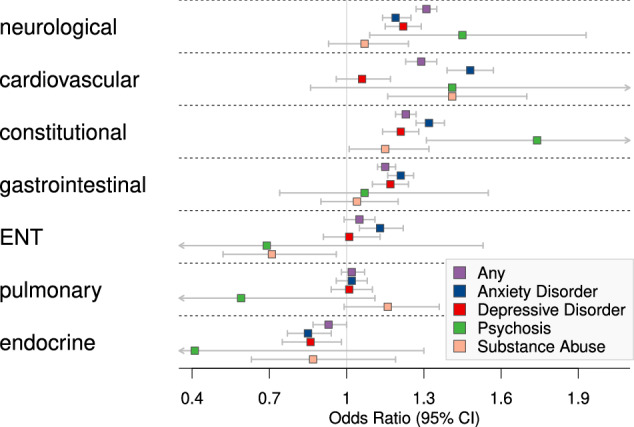
Association of Manifestations to Any Psychiatric Disease. Odds ratios and 95% confidence intervals for the association of features in the seven investigated HPO categories with all newly diagnosed psychiatric disease and the subcategories anxiety disorder, depressive disorder, psychosis, and substance abuse. See also Supplemental Table S2 for complete results including comorbidities and demographics.

We found that there was a significant positive association with any newly diagnosed psychiatric disease for four of the seven investigated HPO categories, with the estimated odds ratio ranging from 1.15 to 1.31 (Fig. 2). Conversely, endocrine PASC-AMs were negatively associated with psychiatric diagnoses with an odds ratio of 0.93 (0.87–0.999, 95% CI). For the subcategory of anxiety disorders, the same four HPO categories and ENT manifestation showed a significant positive association with anxiety disorders. Of the seven HPO categories, three were significantly positively associated with newly diagnosed depressive disorders. Endocrine PASC-AMs had a significant negative association with new-onset depressive disorders. Fewer patients were available with newly diagnosed psychosis, but there was still a significant increase in risk for patients with neurological and constitutional manifestations for being diagnosed with psychosis (Fig. 2). There was a significant positive association with substance abuse and cardiovascular and constitutional PASC-AMs. Interestingly, substance abuse was the only outcome with a significant negative association with ENT.

We performed a sensitivity analysis by limiting the time course in the control group to end at the average time of diagnosis for the group with a psychiatric outcome (72 days). Results showed increased odds ratios in all outcomes resulting in no significant negative associations between outcomes and PASC-AMs (data not shown). Further sensitivity analysis of stratifying inpatients and outpatients, removing patients with any BMI missingness, and varying the definition for the early post-acute phase showed results similar to the main finding.

**Fig. 3:**
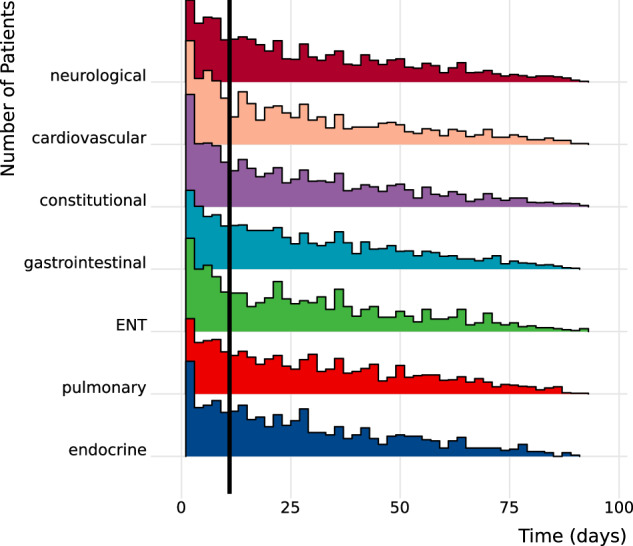
Time Between PASC-AM Diagnosis and Psychiatric Disease. Histogram showing the time between the first PASC-AM and psychiatric disease for each patient and each PASC-AM category. The X-axis shows the number of days between the first recorded PASC-AM for each patient and the recorded onset of psychiatric disease. A black line is drawn at eleven days to indicate data that was excluded in the sensitivity analysis where PASC-AMs occurring within 10 days before the first psychiatric disease were not considered.

To understand the temporal relationship between PASC-AMs and new-onset psychiatric diseases, we selected patients with any PASC-AM and a psychiatric outcome. We then found the time (in days) between each patient’s first PASC-AM in any category and diagnosis of psychiatric disease (Fig. 3). To investigate whether main results were primarily from co-occurring PASC-AMs and psychiatric disease that were merely recorded at the same time, we repeated the main analysis excluding PASC-AMs that were recorded within ten days prior to the new-onset psychiatric disease. Results were negatively shifted from main analysis because of the shortened window for PASC-AMs to be recorded, however, some significant positive associations were retained and differences between findings of categories were largely the same, supporting the temporal relationship of PASC-AMs occurring before psychiatric disease.

**Fig. 4:**
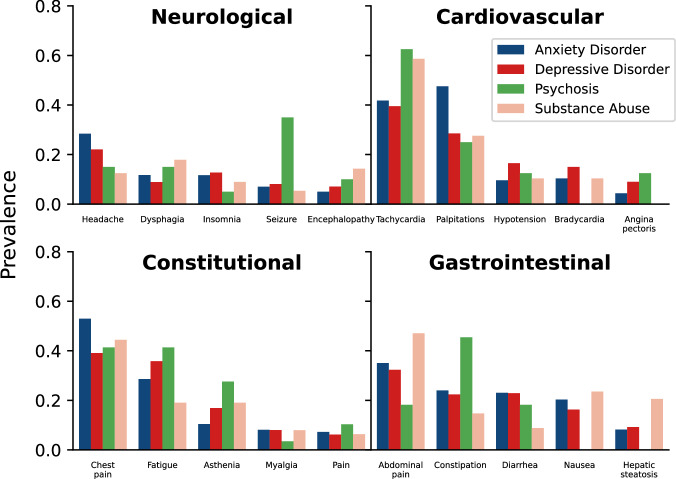
Proportion of Patients with HPO Feature by Category and Outcome. Here we examine the breakdown of individual HPO phenotypic features from symptom categories. The X-axis shows the five most prevalent HPO features from the displayed category. The Y-axis shows the proportion of patients with that feature from the set patients with the indicated HPO feature category and outcome. Significance of each finding was tested using chi-squared test (Supplemental Table S3).

We then investigated the distribution of individual HPO terms for the seven categories described above. We compared counts of the observed HPO terms for each category among patients with diagnoses of new-onset anxiety, depressive disorder, psychosis, and substance abuse. Statistical significance was assessed with a chi-squared test and adjusted for multiple testing. Only patients with at least one HPO term in the category were included in this analysis. To ensure that no patient was in multiple groups, we eliminated the category of all psychiatric diseases and removed patients who simultaneously presented with multiple outcomes, leaving 6,208 patients (Fig. 4, Supplemental Fig. S2 and Table S3).

## Discussion

In this retrospective observational study on a cohort of 2,391,006 individuals following acute COVID-19 infection, we find that constitutional, neurological, gastrointestinal, cardiovascular and ENT PASC-AMs are associated with an increased incidence of new-onset psychiatric disease.

Five studies, including our own previous work, have performed large-scale EHR analyses that show significant and consistent but modest associations between SARS-CoV-2 infection and increased rates of psychiatric disease [4, 5, 9, 23, 24]. Differences in cumulative risk between patients who had COVID-19 and those who had other respiratory tract infections persist for some time following acute infection. However, the risk of new-onset psychiatric disease decreases sharply after the first month after acute SARS-CoV-2 infection and two studies suggest the risk returns to baseline after 120 days [5, 9]. In contrast, a recent study showed that neuropsychiatric sequelae of severe COVID-19 infection were similar to those observed in other severe acute respiratory infections [8]. With the exception of altered mental status, one study showed that neuropsychiatric manifestations are similar between patients with influenza and those with SARS-CoV2 [25].

In the main analysis, endocrine PASC-AMs were associated with a lower frequency of new-onset anxiety and depressive disorders. We hypothesize that this is a result of the shorter sampling window of PASC-AMs for patients with a psychiatric diagnosis compared to controls. That is, PASC-AMs are recorded for controls for the full window of 28–120 days after COVID-19 diagnosis, whereas PASC-AMs occurring at the end of the window (after the psychiatric diagnosis) are not recorded for patients with new-onset psychiatric disease. This leads to a negative bias in the odds ratios because patients with a psychiatric outcome will have fewer PASC-AMs considered when compared to controls whose PASC-AMs occur at the same rate. Sensitivity analysis done by shortening the cutoff for including PASC-AMs in the control group shows a unanimous increase in odds ratios which removes the significant negative association between endocrine PASC-AMs and anxiety and depressive disorders. This supports the hypothesis that the varying time course explains this negative association and suggests that the positive associations we report are conservative.

Our results show that the presence of PASC-AMs in five clinical categories is associated with an increased incidence of newly diagnosed psychiatric disease following COVID-19. There are several possible explanations, including that pathophysiological mechanisms increase the risk of both new-onset psychiatric disease and PASC-AMs or that PASC-AMs themselves increase risk of newly diagnosed psychiatric disease following COVID-19. Our results show different PASC-AM category associations for anxiety disorders, depressive disorders, psychosis, and substance abuse and that individual PASC-AMs within the categories contribute to these associations for each outcome. This may suggest there are several pathophysiological mechanisms in PASC that could explain the heterogeneity of phenotypic presentation [12].

Our dataset is derived from over 76 institutions across the country with 8 million cases of COVID-19, and thus is a representative sample of the COVID-19 positive population in the United States, but inconsistent or incomplete data collection could introduce biases. N3C employs a comprehensive suite of data quality checks to mitigate this problem [26], but residual issues cannot be ruled out. Retrospective analysis of EHR data does not allow any conclusions about pathomechanisms and cannot easily detect misdiagnoses.

In summary, we have shown that the presence of PASC-AMs from any of five clinical categories is associated with increased incidence of newly diagnosed psychiatric disease. This is consistent with the association that has been described between clinical severity of several other chronic diseases (chronic heart failure, chronic kidney disease, chronic hepatitis C, and cancer) and psychiatric disease. In fact, that association is bidirectional: chronic disease is associated with increased rates of psychiatric disease, and psychiatric disease is associated with a higher risk of chronic disease occurrence, severity, or progression [27–30].

Our results have important implications for both individual and public health. Timely and accurate diagnosis of psychiatric conditions has the potential to improve the quality of life for affected individuals; it may be worth systematically testing PASC patients for psychiatric sequelae. The scope of the COVID-19 pandemic is enormous, and it is essential to gain a deeper understanding of the natural history of PASC-related psychiatric diseases and their differential diagnoses to optimize care for affected individuals and institute appropriate public health measures.

### Supplementary information

Online supplemental material

## Data Availability

The data presented in this paper can be accessed upon application to the NCATS N3C Data Enclave at https://covid.cd2h.org/enclave.
