# Supplementary material for: Association of post-COVID phenotypic manifestations with new-onset psychiatric disease
Source: Transl Psychiatry. 2024 Jun 8;14:246. doi: 10.1038/s41398-024-02967-z (PMC11162470; doi:10.1038/s41398-024-02967-z)
Supplement: Supplementary file 1 — Online supplemental material [file 41398_2024_2967_MOESM1_ESM.pdf]

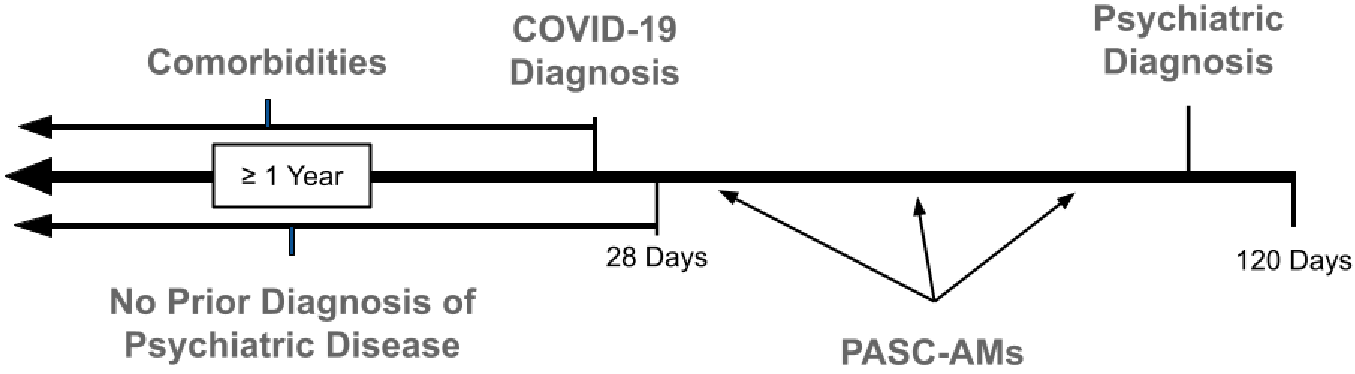

Figure S1. Timeline for measured variables. Every patient was considered with respect to when they were first diagnosed with COVID-19. The pre-COVID phase includes any available records of the patient prior to their COVID-19 diagnosis. Patients whose records did not include at least a year of history prior to COVID-19 diagnosis were removed from the analysis. To focus on patients with a diagnosis of new-onset psychiatric disease, we removed patients with any psychiatric diagnosis before or during their COVID-19 infection (the first 28 days after diagnosis) or anywhere in the pre-COVID phase. Additionally, comorbidities were only considered if they were recorded in the pre-COVID phase. HPO-encoded PASC manifestations were only included if they occurred in the early post-COVID phase and prior to the diagnosis of psychiatric disease (if any). PASC-AMs in the early post-acute phase were used to predict risk of psychiatric disease.

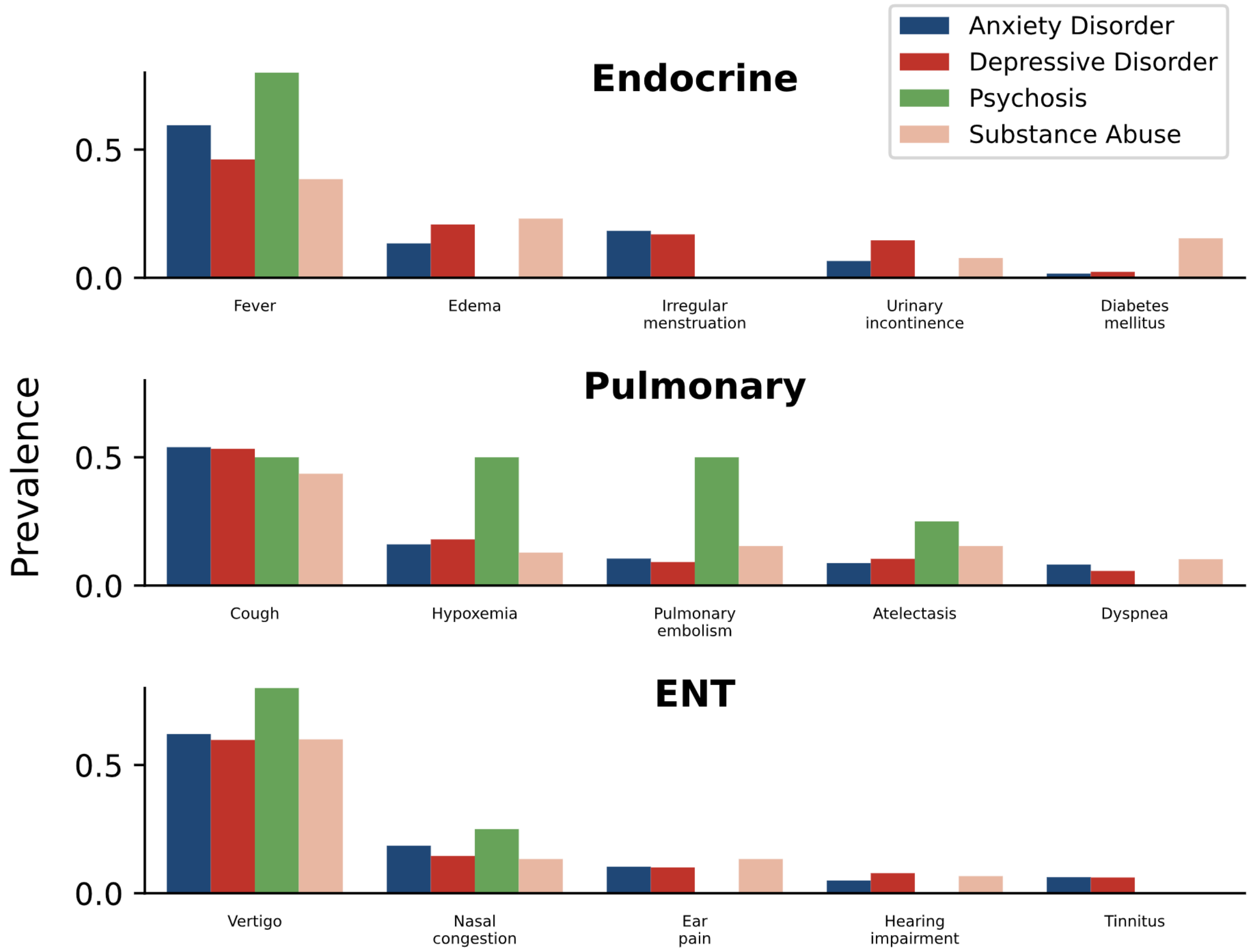

Figure S2. Proportion of Patients with HPO Feature by Category and Outcome. The X-axis shows the five most prevalent HPO features from the displayed category. The Y-axis shows the proportion of patients with that feature from the set of patients with the indicated symptom category and outcome.

Table S1. HPO Terms and Categories. ✓: observed; ∉: no adequate mapping to OMOP; ×: excluded because it describes a psychiatric manifestation; ∅: not observed in the EHR dataset and therefore not used in the analysis.

Id	label	category	status
HP:0001681	Angina pectoris	Cardiovascular	✓
HP:0001662	Bradycardia	Cardiovascular	✓
HP:4000006	Elevated myocardial native T1	Cardiovascular	∉
HP:4000003	Elevated myocardial native T2	Cardiovascular	∉
HP:0000822	Hypertension	Cardiovascular	✓
HP:0002615	Hypotension	Cardiovascular	✓
HP:0410173	Increased circulating troponin I concentration	Cardiovascular	∉
HP:0410174	Increased circulating troponin T concentration	Cardiovascular	∉
HP:0031862	Increased heart rate variability	Cardiovascular	∉
HP:0033755	Increased left ventricular end-diastolic volume	Cardiovascular	∉
HP:4000004	Myocardial late gadolinium enhancement	Cardiovascular	∉
HP:0012819	Myocarditis	Cardiovascular	✓
HP:0001962	Palpitations	Cardiovascular	✓
HP:0001698	Pericardial effusion	Cardiovascular	✓
HP:4000005	Pericardial late gadolinium enhancement	Cardiovascular	∉
HP:0012664	Reduced ejection fraction	Cardiovascular	∉
HP:0001297	Stroke	Cardiovascular	✓
HP:0001279	Syncope	Cardiovascular	✓
HP:0001649	Tachycardia	Cardiovascular	✓
HP:0004936	Venous thrombosis	Cardiovascular	✓
HP:0002829	Arthralgia	Constitutional	✓
HP:0001369	Arthritis	Constitutional	✓
HP:0025406	Asthenia	Constitutional	✓
HP:0033047	Body ache	Constitutional	∉
HP:0002653	Bone pain	Constitutional	✓
HP:0100749	Chest pain	Constitutional	✓
HP:0031352	Chest tightness	Constitutional	✓
HP:0025143	Chills	Constitutional	∉
HP:0033850	Coldness	Constitutional	∉
HP:0002355	Difficulty walking	Constitutional	✓
HP:0033665	Diminished health-related quality of life	Constitutional	∉
HP:0033667	Diminished mental health	Constitutional	∉
HP:0033666	Diminished physical functioning	Constitutional	∉
HP:0003546	Exercise intolerance	Constitutional	✓
HP:0012378	Fatigue	Constitutional	✓
HP:0033675	Frailty	Constitutional	∉
HP:0031059	Impaired ability to bathe oneself	Constitutional	∉
HP:0031060	Impaired ability to dress oneself	Constitutional	∉
HP:0031058	Impairment of activities of daily living	Constitutional	∉
HP:0033746	Intrascapular pain	Constitutional	∉
HP:0009763	Limb pain	Constitutional	✓
HP:0033834	Malaise	Constitutional	∉
HP:0003326	Myalgia	Constitutional	✓
HP:0033345	Neuralgia	Constitutional	∉

Continued on next page

Table S1 – continued from previous page

Id	label	category	status
HP:0030166	Night sweats	Constitutional	✓
HP:0033695	Occupational disability	Constitutional	∉
HP:0012531	Pain	Constitutional	✓
HP:0030973	Postexertional malaise	Constitutional	×
HP:0025144	Shivering	Constitutional	✓
HP:0025258	Stiff neck	Constitutional	∉
HP:0001824	Weight loss	Constitutional	∉
HP:0000217	Xerostomia	Constitutional	✓
HP:0012213	Decreased glomerular filtration rate	Endocrine	∉
HP:0000819	Diabetes mellitus	Endocrine	✓
HP:0000969	Edema	Endocrine	✓
HP:0030014	Female sexual dysfunction	Endocrine	∉
HP:0001945	Fever	Endocrine	✓
HP:0002046	Heat intolerance	Endocrine	✓
HP:0002045	Hypothermia	Endocrine	✓
HP:0000858	Irregular menstruation	Endocrine	✓
HP:0011134	Low-grade fever	Endocrine	✓
HP:0040307	Male sexual dysfunction	Endocrine	✓
HP:0000132	Menorrhagia	Endocrine	✓
HP:0001733	Pancreatitis	Endocrine	✓
HP:0033840	Postmenopausal bleeding	Endocrine	∉
HP:0001954	Recurrent fever	Endocrine	✓
HP:0000083	Renal insufficiency	Endocrine	✓
HP:0005968	Temperature instability	Endocrine	∉
HP:0033839	Testicular pain	Endocrine	∉
HP:0000020	Urinary incontinence	Endocrine	✓
HP:0001618	Dysphonia	Ent	∉
HP:0030766	Ear pain	Ent	✓
HP:0000365	Hearing impairment	Ent	✓
HP:0010780	Hyperacusis	Ent	✓
HP:0001742	Nasal congestion	Ent	✓
HP:0033050	Pharyngalgia	Ent	✓
HP:0008629	Pulsatile tinnitus	Ent	✓
HP:0012384	Rhinitis	Ent	✓
HP:0000360	Tinnitus	Ent	✓
HP:0002321	Vertigo	Ent	✓
HP:0000618	Blindness	Eye	✓
HP:0000622	Blurred vision	Eye	✓
HP:0000509	Conjunctivitis	Eye	✓
HP:0000651	Diplopia	Eye	✓
HP:0000640	Gaze-evoked nystagmus	Eye	×
HP:0001097	Keratoconjunctivitis sicca	Eye	✓
HP:0200026	Ocular pain	Eye	✓
HP:0033841	Ocular pruritus	Eye	∉
HP:0007994	Peripheral visual field loss	Eye	×
HP:0000613	Photophobia	Eye	✓
HP:0025337	Red eye	Eye	✓
		Continued on next page	

Table S1 – continued from previous page

Id	label	category	status
HP:0000572	Visual loss	Eye	×
HP:0100832	Vitreous floaters	Eye	✓
HP:0002027	Abdominal pain	Gastrointestinal	✓
HP:0011458	Abdominal symptom	Gastrointestinal	∉
HP:0002039	Anorexia	Gastrointestinal	∉
HP:0002607	Bowel incontinence	Gastrointestinal	✓
HP:0002019	Constipation	Gastrointestinal	✓
HP:0002014	Diarrhea	Gastrointestinal	✓
HP:0033842	Early satiety	Gastrointestinal	∉
HP:0002592	Gastric ulcer	Gastrointestinal	✓
HP:0002020	Gastroesophageal reflux	Gastrointestinal	✓
HP:0002578	Gastroparesis	Gastrointestinal	✓
HP:0001397	Hepatic steatosis	Gastrointestinal	✓
HP:0012115	Hepatitis	Gastrointestinal	✓
HP:0002240	Hepatomegaly	Gastrointestinal	✓
HP:0004395	Malnutrition	Gastrointestinal	✓
HP:0002018	Nausea	Gastrointestinal	✓
HP:0033757	Pancreatic steatosis	Gastrointestinal	∉
HP:0004396	Poor appetite	Gastrointestinal	∉
HP:0001744	Splenomegaly	Gastrointestinal	✓
HP:0002013	Vomiting	Gastrointestinal	✓
HP:0100845	Anaphylactic shock	Immunology	✓
HP:0032069	Anti-thyroglobulin antibody positivity	Immunology	∉
HP:0025379	Anti-thyroid peroxidase antibody positivity	Immunology	∉
HP:0003493	Antinuclear antibody positivity	Immunology	✓
HP:0002716	Lymphadenopathy	Immunology	✓
HP:0001888	Lymphopenia	Immunology	✓
HP:0012053	Decreased circulating calcifediol concentration	Laboratory	∉
HP:0031964	Elevated circulating alanine aminotransferase concentration	Laboratory	∉
HP:0003155	Elevated circulating alkaline phosphatase concentration	Laboratory	✓
HP:0031956	Elevated circulating aspartate aminotransferase concentration	Laboratory	∉
HP:0011227	Elevated circulating C-reactive protein concentration	Laboratory	✓
HP:0003236	Elevated circulating creatine kinase concentration	Laboratory	∉
HP:0003259	Elevated circulating creatinine concentration	Laboratory	✓
HP:0033106	Elevated circulating D-dimer concentration	Laboratory	∉
HP:0033833	Elevated circulating soluble CD25 concentration	Laboratory	∉
HP:0002925	Elevated circulating thyroid-stimulating hormone concentration	Laboratory	✓
HP:0003565	Elevated erythrocyte sedimentation rate	Laboratory	✓
HP:0030948	Elevated gamma-glutamyltransferase level	Laboratory	∉
HP:0003074	Hyperglycemia	Laboratory	✓
HP:0002901	Hypocalcemia	Laboratory	✓
HP:0011900	Hypofibrinogenemia	Laboratory	×
HP:0001943	Hypoglycemia	Laboratory	✓
HP:0002148	Hypophosphatemia	Laboratory	✓
HP:0003281	Increased circulating ferritin concentration	Laboratory	∉
HP:0030783	Increased circulating interleukin 6	Laboratory	∉
HP:0025435	Increased circulating lactate dehydrogenase concentration	Laboratory	∉

Continued on next page

Table S1 – continued from previous page

Id	label	category	status
HP:0031185	Increased circulating NT-proBNP concentration	Laboratory	∉
HP:0032308	Increased circulating procalcitonin concentration	Laboratory	∉
HP:0001873	Thrombocytopenia	Laboratory	✓
HP:0033747	Abnormal exteroceptive sensation	Neurological	∉
HP:0031826	Abnormal reflex	Neurological	✓
HP:0100022	Abnormality of movement	Neurological	✓
HP:0041051	Ageusia	Neurological	✓
HP:0000718	Aggressive behavior	Neurological	∅
HP:0010524	Agnosia	Neurological	✓
HP:0030784	Anomic aphasia	Neurological	×
HP:0000458	Anosmia	Neurological	✓
HP:0033689	Anterograde memory impairment	Neurological	∉
HP:0000739	Anxiety	Neurological	∅
HP:0000741	Apathy	Neurological	∅
HP:0002381	Aphasia	Neurological	✓
HP:0001251	Ataxia	Neurological	✓
HP:0007018	Attention deficit hyperactivity disorder	Neurological	∅
HP:0008765	Auditory hallucinations	Neurological	∅
HP:0003487	Babinski sign	Neurological	✓
HP:0033849	Bilingual aphasia	Neurological	∉
HP:0002067	Bradykinesia	Neurological	✓
HP:0031843	Bradyphrenia	Neurological	×
HP:0033630	Brain fog	Neurological	∉
HP:0100543	Cognitive impairment	Neurological	∉
HP:0001289	Confusion	Neurological	✓
HP:0000746	Delusions	Neurological	∅
HP:0000716	Depression	Neurological	∅
HP:0031987	Diminished ability to concentrate	Neurological	∅
HP:0001260	Dysarthria	Neurological	✓
HP:0001310	Dysmetria	Neurological	✓
HP:0002015	Dysphagia	Neurological	✓
HP:0033838	Dysphoria	Neurological	∉
HP:0001332	Dystonia	Neurological	✓
HP:0000712	Emotional lability	Neurological	∉
HP:0001298	Encephalopathy	Neurological	✓
HP:0031844	Euphoria	Neurological	×
HP:0002427	Expressive aphasia	Neurological	✓
HP:0007209	Facial paralysis	Neurological	∉
HP:0000743	Frontal release signs	Neurological	∉
HP:0001288	Gait disturbance	Neurological	∉
HP:0000738	Hallucinations	Neurological	∅
HP:0030237	Hand muscle weakness	Neurological	✓
HP:0002315	Headache	Neurological	✓
HP:0100963	Hyperesthesia	Neurological	✓
HP:0002487	Hyperkinetic movements	Neurological	✓
HP:0033748	Hypoesthesia	Neurological	∉
HP:0000224	Hypogeusia	Neurological	×

Continued on next page

Table S1 – continued from previous page

Id	label	category	status
HP:0004409	Hyposmia	Neurological	✓
HP:0001252	Hypotonia	Neurological	∉
HP:0033051	Impaired executive functioning	Neurological	✓
HP:0100710	Impulsivity	Neurological	∉
HP:0100785	Insomnia	Neurological	✓
HP:0000737	Irritability	Neurological	∅
HP:0033688	Long term memory impairment	Neurological	∉
HP:0031355	Maintenance insomnia	Neurological	∉
HP:0100754	Mania	Neurological	∅
HP:0002354	Memory impairment	Neurological	✓
HP:0002076	Migraine	Neurological	✓
HP:0003394	Muscle spasm	Neurological	✓
HP:0001324	Muscle weakness	Neurological	✓
HP:0001278	Orthostatic hypotension	Neurological	✓
HP:0025269	Panic attack	Neurological	∅
HP:0031249	Parageusia	Neurological	✓
HP:0003401	Paresthesia	Neurological	✓
HP:0001300	Parkinsonism	Neurological	✓
HP:0033847	Phantageusia	Neurological	∉
HP:0033693	Phantosmia	Neurological	∉
HP:0002183	Phonophobia	Neurological	∅
HP:0001959	Polydipsia	Neurological	∉
HP:0001271	Polyneuropathy	Neurological	✓
HP:0033676	Posttraumatic stress symptom	Neurological	∉
HP:0033691	Procedural memory loss	Neurological	∉
HP:0033848	Receptive aphasia	Neurological	∉
HP:0012452	Restless legs	Neurological	✓
HP:0002063	Rigidity	Neurological	∉
HP:0001250	Seizure	Neurological	✓
HP:0033845	Sense of impending doom	Neurological	∉
HP:0000736	Short attention span	Neurological	∅
HP:0033687	Short term memory impairment	Neurological	∉
HP:0003202	Skeletal muscle atrophy	Neurological	✓
HP:0010535	Sleep apnea	Neurological	✓
HP:0002360	Sleep disturbance	Neurological	✓
HP:0031354	Sleep onset insomnia	Neurological	∉
HP:0001350	Slurred speech	Neurological	✓
HP:0003474	Somatic sensory dysfunction	Neurological	✓
HP:0001257	Spasticity	Neurological	✓
HP:0031589	Suicidal ideation	Neurological	∉
HP:0033844	Tachyphrenia	Neurological	∉
HP:0033694	Tactile hallucination	Neurological	∉
HP:0033705	Tearfulness	Neurological	∉
HP:0031356	Terminal insomnia	Neurological	×
HP:0001337	Tremor	Neurological	✓
HP:0012799	Unilateral facial palsy	Neurological	∉
HP:0002367	Visual hallucinations	Neurological	∅

Continued on next page

Table S1 – continued from previous page

Id	label	category	status
HP:0031983	Abnormal pulmonary thoracic imaging finding	Pulmonary	∉
HP:0006536	Airway obstruction	Pulmonary	∉
HP:0100750	Atelectasis	Pulmonary	✓
HP:0002110	Bronchiectasis	Pulmonary	✓
HP:0025180	Centrilobular ground-glass opacification on pulmonary HRCT	Pulmonary	∉
HP:0012735	Cough	Pulmonary	✓
HP:0033659	Crazy-paving pattern	Pulmonary	∉
HP:0045051	Decreased DLCO	Pulmonary	∉
HP:0033760	Decreased maximal oxygen uptake	Pulmonary	∉
HP:0033773	Decreased RV/TLC ratio	Pulmonary	∉
HP:0002094	Dyspnea	Pulmonary	✓
HP:0002875	Exertional dyspnea	Pulmonary	✓
HP:0025179	Ground-glass opacification	Pulmonary	∉
HP:0002105	Hemoptysis	Pulmonary	✓
HP:0012418	Hypoxemia	Pulmonary	✓
HP:0033709	Increased sputum production	Pulmonary	∉
HP:0030879	Interlobular septal thickening	Pulmonary	∉
HP:0031246	Nonproductive cough	Pulmonary	✓
HP:0030874	Oxygen desaturation on exertion	Pulmonary	∉
HP:0032177	Parenchymal consolidation	Pulmonary	∉
HP:0031944	Pleural thickening	Pulmonary	✓
HP:0033771	Pleuritic chest pain	Pulmonary	∉
HP:0002102	Pleuritis	Pulmonary	✓
HP:0031245	Productive cough	Pulmonary	✓
HP:0032446	Pulmonary bulla	Pulmonary	∉
HP:0002204	Pulmonary embolism	Pulmonary	✓
HP:0002206	Pulmonary fibrosis	Pulmonary	✓
HP:0033711	Pulmonary interstitial thickening	Pulmonary	∉
HP:0030877	Reduced FEV1/FVC ratio	Pulmonary	∉
HP:0032342	Reduced forced expiratory volume in one second	Pulmonary	∉
HP:0032341	Reduced forced vital capacity	Pulmonary	∉
HP:0033750	Reduced functional residual capacity	Pulmonary	∉
HP:0033753	Reduced residual volume	Pulmonary	∉
HP:0033169	Reduced total lung capacity	Pulmonary	∉
HP:0033710	Rest dyspnea	Pulmonary	∉
HP:0002091	Restrictive ventilatory defect	Pulmonary	✓
HP:0025390	Reticular pattern on pulmonary HRCT	Pulmonary	∉
HP:0031417	Rhinorrhea	Pulmonary	✓
HP:0030831	Rhonchi	Pulmonary	✓
HP:0025095	Sneeze	Pulmonary	✓
HP:0033609	Solid pulmonary nodule	Pulmonary	∉
HP:0033702	Subpleural curvilinear line	Pulmonary	∉
HP:0033610	Subsolid pulmonary nodule	Pulmonary	∉
HP:0002789	Tachypnea	Pulmonary	∉
HP:0030828	Wheezing	Pulmonary	✓
HP:0001596	Alopecia	Skin	✓
HP:0011971	Dermatographic urticaria	Skin	✓

Continued on next page

Table S1 – continued from previous page

Id	label	category	status
HP:0031284	Flushing	Skin	∉
HP:0001808	Fragile nails	Skin	∉
HP:0000975	Hyperhidrosis	Skin	✓
HP:0000967	Petechiae	Skin	✓
HP:0000989	Pruritus	Skin	✓
HP:0033696	Pseudo-chilblain	Skin	∉
HP:0040189	Scaling skin	Skin	✓
HP:0000988	Skin rash	Skin	✓

Table S2. Logistic Regression for All Factors and Outcomes. Odds ratios, 95% confidence intervals, and p-values for the association of features in all factors and outcomes.

	Any Psychiatric Disease		Anxiety Disorder		Depressive Disorder		Psychosis		Substance Abuse	
Predictor	OR (CI)	p-val	OR (CI)	p-val	OR (CI)	p-val	OR (CI)	p-val	OR (CI)	p-val
(Intercept)	0.03 (0.02-0.03)	<0.05	0.02 (0.01-0.02)	<0.05	0.01 (0.01-0.01)	<0.05	0 (0-0)	<0.05	0 (0-0)	<0.05
neurological	1.31 (1.27-1.35)	<0.05	1.19 (1.14-1.25)	<0.05	1.22 (1.15-1.29)	<0.05	1.45 (1.09-1.93)	<0.05	1.07 (0.93-1.24)	0.33
cardiovascular	1.29 (1.23-1.35)	<0.05	1.48 (1.39-1.57)	<0.05	1.06 (0.96-1.17)	0.23	1.41 (0.86-2.3)	0.17	1.41 (1.16-1.7)	<0.05
constitutional	1.23 (1.19-1.27)	<0.05	1.32 (1.27-1.38)	<0.05	1.21 (1.14-1.28)	<0.05	1.74 (1.31-2.3)	<0.05	1.15 (1.01-1.32)	<0.05
gastrointestinal	1.15 (1.12-1.19)	<0.05	1.21 (1.16-1.26)	<0.05	1.17 (1.1-1.24)	<0.05	1.07 (0.74-1.55)	0.71	1.04 (0.9-1.2)	0.59
ENT	1.05 (0.99-1.11)	0.083	1.13 (1.05-1.22)	<0.05	1.01 (0.91-1.13)	0.83	0.69 (0.31-1.53)	0.37	0.71 (0.52-0.96)	<0.05
pulmonary	1.02 (0.98-1.07)	0.25	1.02 (0.96-1.08)	0.56	1.01 (0.94-1.1)	0.75	0.59 (0.32-1.11)	0.1	1.16 (0.99-1.36)	0.069
endocrine	0.93 (0.87-1)	<0.05	0.85 (0.77-0.94)	<0.05	0.86 (0.75-0.98)	<0.05	0.41 (0.13-1.3)	0.13	0.87 (0.63-1.19)	0.37
BMI under 20	0.81 (0.75-0.88)	<0.05	0.56 (0.5-0.63)	<0.05	0.4 (0.33-0.47)	<0.05	0.39 (0.16-0.95)	<0.05	0.35 (0.26-0.46)	<0.05
BMI 20 to 25	0.92 (0.86-0.98)	<0.05	0.88 (0.8-0.97)	<0.05	0.78 (0.71-0.85)	<0.05	0.97 (0.58-1.61)	0.89	0.96 (0.8-1.14)	0.6
BMI 25 to 30	0.89 (0.8-0.97)	<0.05	0.88 (0.78-0.98)	<0.05	0.81 (0.73-0.91)	<0.05	1.28 (0.81-2.04)	0.28	1.05 (0.86-1.28)	0.61
BMI 30 to 35	0.88 (0.79-0.98)	<0.05	0.88 (0.78-0.99)	<0.05	0.84 (0.74-0.94)	<0.05	1.19 (0.74-1.9)	0.47	1 (0.81-1.23)	1
BMI 35 to 40	0.94 (0.87-1.03)	0.15	0.96 (0.86-1.07)	0.38	0.93 (0.83-1.04)	0.18	1.11 (0.61-2.02)	0.72	0.99 (0.8-1.22)	0.92
smoking status	1.52 (1.44-1.6)	<0.05	1.45 (1.35-1.55)	<0.05	1.45 (1.32-1.59)	<0.05	2.66 (1.68-4.21)	<0.05	3.65 (3.17-4.2)	<0.05
gender FEMALE	1 (1-1)	REF	1 (1-1)	REF	1 (1-1)	REF	1 (1-1)	REF	1 (1-1)	REF
gender MALE	0.65 (0.64-0.67)	<0.05	0.49 (0.48-0.51)	<0.05	0.46 (0.45-0.48)	<0.05	1.29 (1.06-1.58)	<0.05	1.79 (1.65-1.94)	<0.05
race ethnicity White Non Hispanic	1 (1-1)	REF	1 (1-1)	REF	1 (1-1)	REF	1 (1-1)	REF	1 (1-1)	REF
race ethnicity Hispanic or Latino Any Race	1.01 (0.98-1.04)	0.46	0.88 (0.84-0.92)	<0.05	0.95 (0.9-1)	<0.05	1.41 (1.04-1.91)	<0.05	0.91 (0.8-1.04)	0.19
race ethnicity Black or African American Non Hispanic	0.91 (0.88-0.93)	<0.05	0.72 (0.69-0.75)	<0.05	0.77 (0.73-0.81)	<0.05	2.39 (1.87-3.04)	<0.05	1.47 (1.33-1.62)	<0.05
race ethnicity Unknown	0.83 (0.79-0.87)	<0.05	0.77 (0.72-0.82)	<0.05	0.69 (0.63-0.76)	<0.05	1.42 (0.89-2.25)	0.14	0.77 (0.62-0.97)	<0.05
race ethnicity Asian Non Hispanic	0.69 (0.65-0.74)	<0.05	0.61 (0.55-0.67)	<0.05	0.62 (0.55-0.7)	<0.05	1.2 (0.63-2.29)	0.57	0.65 (0.48-0.88)	<0.05
race ethnicity Native Hawaiian or Other Pacific Islander Non Hispanic	0.69 (0.56-0.85)	<0.05	0.54 (0.4-0.75)	<0.05	0.67 (0.47-0.96)	<0.05	null	null	1.12 (0.6-2.1)	0.72
race ethnicity Other Non Hispanic	0.58 (0.53-0.64)	<0.05	0.47 (0.41-0.54)	<0.05	0.54 (0.45-0.64)	<0.05	1.25 (0.56-2.83)	0.59	0.46 (0.28-0.76)	<0.05
chronic resp	1.29 (1.25-1.33)	<0.05	1.31 (1.26-1.37)	<0.05	1.21 (1.14-1.27)	<0.05	1.08 (0.77-1.51)	0.65	1.16 (1.04-1.31)	<0.05
diabetes2	1.05 (1.02-1.09)	<0.05	0.94 (0.89-0.99)	<0.05	1.21 (1.14-1.28)	<0.05	1.44 (1.03-2.03)	<0.05	0.9 (0.79-1.03)	0.13
nicotine dependence	1.25 (1.19-1.31)	<0.05	1.25 (1.17-1.35)	<0.05	1.19 (1.08-1.3)	<0.05	0.78 (0.45-1.35)	0.37	2.72 (2.35-3.13)	<0.05
other liver disease	1.15 (1.09-1.23)	<0.05	1.09 (1-1.19)	0.063	1.03 (0.92-1.16)	0.59	1.96 (1.1-3.49)	<0.05	1.6 (1.3-1.96)	<0.05
hypertension	1.13 (1.1-1.16)	<0.05	1.07 (1.03-1.11)	<0.05	1.11 (1.05-1.16)	<0.05	0.91 (0.68-1.22)	0.53	1.17 (1.05-1.3)	<0.05
non ischemic heart disease	1.1 (1.07-1.14)	<0.05	0.98 (0.93-1.02)	0.32	1.04 (0.98-1.1)	0.18	1.28 (0.92-1.8)	0.15	1.1 (0.97-1.25)	0.14
neoplasm	1.07 (1.04-1.1)	<0.05	1.09 (1.05-1.13)	<0.05	0.95 (0.9-0.99)	<0.05	0.57 (0.41-0.8)	<0.05	0.87 (0.78-0.97)	<0.05
non hypertensive chronic kidney disease	1.07 (1.01-1.13)	<0.05	0.81 (0.73-0.89)	<0.05	1.03 (0.93-1.15)	0.57	1.29 (0.74-2.27)	0.37	1.13 (0.9-1.41)	0.28
hepatic steatosis	1.02 (0.96-1.09)	0.47	1.08 (0.99-1.18)	0.1	1.02 (0.91-1.15)	0.68	0.32 (0.1-1.02)	0.054	1.24 (0.98-1.57)	0.08
hypertensive kidney disease	1.01 (0.93-1.1)	0.76	1.05 (0.91-1.2)	0.51	1.03 (0.89-1.19)	0.72	1.02 (0.47-2.19)	0.97	0.93 (0.68-1.25)	0.62
ischemic heart disease	0.99 (0.93-1.04)	0.6	0.95 (0.88-1.04)	0.29	1.06 (0.96-1.18)	0.24	0.66 (0.35-1.27)	0.21	0.94 (0.77-1.15)	0.53

Table S3. Chi-Square Analysis of Differential Rates of HPO Terms Between Outcomes: In this analysis we perform a chi-square test to determine whether individual HPO terms are present at different rates in the groups of patients with each outcome. Count gives the total number of patients with the given term. P-val provides the Bonferroni corrected p-values for whether the difference between outcome groups is significant. Category provides the PASC-AM category that the HPO term was included in for the main analysis.

HPO Term	count	p-val (adj)	category
Chest pain (HP:0100749)	34374	2.16×10^{-165}	constitutional
Tachycardia (HP:0001649)	10142	1.42×10^{-120}	cardiovascular
Palpitations (HP:0001962)	13438	2.45×10^{-77}	cardiovascular
Asthenia (HP:0025406)	8177	4.16×10^{-56}	constitutional
Confusion (HP:0001289)	1272	5.69×10^{-53}	neurological
Vertigo (HP:0002321)	15868	3.68×10^{-50}	ENT
Encephalopathy (HP:0001298)	2239	1.11×10^{-40}	neurological
Fatigue (HP:0012378)	28382	1.33×10^{-39}	constitutional
Nausea (HP:0002018)	10177	2.09×10^{-36}	gastrointestinal
Abdominal pain (HP:0002027)	22718	4.28×10^{-34}	gastrointestinal
Headache (HP:0002315)	18372	4.53×10^{-32}	neurological
Hypoxemia (HP:0012418)	6244	6.30×10^{-27}	pulmonary
Atelectasis (HP:0100750)	2961	9.96×10^{-27}	pulmonary
Insomnia (HP:0100785)	6964	6.56×10^{-23}	neurological
Diarrhea (HP:0002014)	14770	5.01×10^{-22}	gastrointestinal
Pancreatitis (HP:0001733)	< 20	5.95×10^{-20}	endocrine
Seizure (HP:0001250)	5392	9.30×10^{-20}	neurological
Constipation (HP:0002019)	15945	1.24×10^{-19}	gastrointestinal
Hypotension (HP:0002615)	3340	4.74×10^{-16}	cardiovascular
Dysphagia (HP:0002015)	7397	5.54×10^{-16}	neurological
Paresthesia (HP:0003401)	6230	4.15×10^{-11}	neurological
Dysarthria (HP:0001260)	392	5.33×10^{-11}	neurological
Thrombocytopenia (HP:0001873)	5890	1.19×10^{-10}	laboratory
Myalgia (HP:0003326)	7413	1.17×10^{-8}	constitutional
Shivering (HP:0025144)	1420	3.36×10^{-8}	constitutional
Hepatitis (HP:0012115)	225	3.51×10^{-8}	gastrointestinal
Memory impairment (HP:0002354)	2755	3.82×10^{-8}	neurological
Migraine (HP:0002076)	5067	6.84×10^{-8}	neurological
Hypoglycemia (HP:0001943)	1339	1.64×10^{-7}	laboratory
Pain (HP:0012531)	6961	2.22×10^{-6}	constitutional
Tremor (HP:0001337)	1924	3.22×10^{-6}	neurological
Hypocalcemia (HP:0002901)	1433	5.79×10^{-6}	laboratory
Dyspnea (HP:0002094)	3835	9.41×10^{-6}	pulmonary
Gastroparesis (HP:0002578)	851	5.77×10^{-5}	gastrointestinal
Pulmonary embolism (HP:0002204)	5318	1.06×10^{-4}	pulmonary
Pleuritis (HP:0002102)	763	4.05×10^{-4}	pulmonary
Hyperglycemia (HP:0003074)	8621	4.21×10^{-4}	laboratory
Hyperkinetic movements (HP:0002487)	5306	1.33×10^{-3}	neurological
Pulmonary fibrosis (HP:0002206)	1637	1.34×10^{-3}	pulmonary
Aphasia (HP:0002381)	545	2.67×10^{-3}	neurological
Pericardial effusion (HP:0001698)	41	3.01×10^{-3}	cardiovascular

Continued on next page

Table S3 – continued from previous page

HPO Term	count	p-val (adj)	category
Angina pectoris (HP:0001681)	2413	4.90×10^{-3}	cardiovascular
Arthralgia (HP:0002829)	5852	5.51×10^{-3}	constitutional
Polyneuropathy (HP:0001271)	5613	5.55×10^{-3}	neurological
Antinuclear antibody positivity (HP:0003493)	46	9.40×10^{-3}	immunology
Bradycardia (HP:0001662)	5597	2.42×10^{-2}	cardiovascular
Hemoptysis (HP:0002105)	791	3.33×10^{-2}	pulmonary
Limb pain (HP:0009763)	3140	3.64×10^{-2}	constitutional
Splenomegaly (HP:0001744)	912	3.97×10^{-2}	gastrointestinal
Lymphadenopathy (HP:0002716)	1475	4.40×10^{-2}	immunology
Sleep apnea (HP:0010535)	5767	8.90×10^{-2}	neurological
Tinnitus (HP:0000360)	1873	1.26×10^{-1}	ENT
Restless legs (HP:0012452)	2428	1.41×10^{-1}	neurological
Productive cough (HP:0031245)	< 20	1.49×10^{-1}	pulmonary
Syncope (HP:0001279)	954	1.79×10^{-1}	cardiovascular
Slurred speech (HP:0001350)	261	1.94×10^{-1}	neurological
Nasal congestion (HP:0001742)	12028	2.69×10^{-1}	ENT
Hepatic steatosis (HP:0001397)	7222	4.17×10^{-1}	gastrointestinal
Elevated circulating creatinine concentration (HP:0003259)	71	6.81×10^{-1}	laboratory
Irregular menstruation (HP:0000858)	4565	7.06×10^{-1}	endocrine
Chest tightness (HP:0031352)	64	9.26×10^{-1}	constitutional
Abnormal reflex (HP:0031826)	182	1.0	neurological
Abnormality of movement (HP:0100022)	174	1.0	neurological
Ageusia (HP:0041051)	< 20	1.0	neurological
Agnosia (HP:0010524)	38	1.0	neurological
Alopecia (HP:0001596)	113	1.0	skin
Anaphylactic shock (HP:0100845)	316	1.0	immunology
Anosmia (HP:0000458)	777	1.0	neurological
Arthritis (HP:0001369)	256	1.0	constitutional
Ataxia (HP:0001251)	391	1.0	neurological
Babinski sign (HP:0003487)	< 20	1.0	neurological
Blindness (HP:0000618)	< 20	1.0	eye
Blurred vision (HP:0000622)	76	1.0	eye
Bone pain (HP:0002653)	< 20	1.0	constitutional
Bowel incontinence (HP:0002607)	815	1.0	gastrointestinal
Bradykinesia (HP:0002067)	< 20	1.0	neurological
Bronchiectasis (HP:0002110)	1455	1.0	pulmonary
Conjunctivitis (HP:0000509)	1635	1.0	eye
Cough (HP:0012735)	42031	1.0	pulmonary
Dermatographic urticaria (HP:0011971)	219	1.0	skin
Diabetes mellitus (HP:0000819)	641	1.0	endocrine
Difficulty walking (HP:0002355)	1864	1.0	constitutional
Diplopia (HP:0000651)	743	1.0	eye
Dystonia (HP:0001332)	191	1.0	neurological
Ear pain (HP:0030766)	4730	1.0	ENT
Edema (HP:0000969)	5007	1.0	endocrine

Continued on next page

Table S3 – continued from previous page

HPO Term	count	p-val (adj)	category
Elevated circulating alkaline phosphatase concentration (HP:0003155)	< 20	1.0	laboratory
Elevated circulating C reactive protein concentration (HP:0011227)	108	1.0	laboratory
Elevated circulating thyroid stimulating hormone concentration (HP:0002925)	41	1.0	laboratory
Elevated erythrocyte sedimentation rate (HP:0003565)	551	1.0	laboratory
Exercise intolerance (HP:0003546)	< 20	1.0	constitutional
Exertional dyspnea (HP:0002875)	755	1.0	pulmonary
Expressive aphasia (HP:0002427)	< 20	1.0	neurological
Fever (HP:0001945)	22140	1.0	endocrine
Gastric ulcer (HP:0002592)	< 20	1.0	gastrointestinal
Gastroesophageal reflux (HP:0002020)	5375	1.0	gastrointestinal
Hand muscle weakness (HP:0030237)	< 20	1.0	neurological
Hearing impairment (HP:0000365)	2725	1.0	ENT
Hepatomegaly (HP:0002240)	1066	1.0	gastrointestinal
Hyperacusis (HP:0010780)	63	1.0	ENT
Hyperesthesia (HP:0100963)	56	1.0	neurological
Hyperhidrosis (HP:0000975)	31	1.0	skin
Hypertension (HP:0000822)	932	1.0	cardiovascular
Hypophosphatemia (HP:0002148)	22	1.0	laboratory
Hyposmia (HP:0004409)	< 20	1.0	neurological
Hypothermia (HP:0002045)	89	1.0	endocrine
Keratoconjunctivitis sicca (HP:0001097)	6994	1.0	eye
Low grade fever (HP:0011134)	< 20	1.0	endocrine
Lymphopenia (HP:0001888)	391	1.0	immunology
Male sexual dysfunction (HP:0040307)	< 20	1.0	endocrine
Malnutrition (HP:0004395)	528	1.0	gastrointestinal
Menorrhagia (HP:0000132)	484	1.0	endocrine
Muscle spasm (HP:0003394)	70	1.0	neurological
Muscle weakness (HP:0001324)	3600	1.0	neurological
Myocarditis (HP:0012819)	209	1.0	cardiovascular
Night sweats (HP:0030166)	< 20	1.0	constitutional
Nonproductive cough (HP:0031246)	< 20	1.0	pulmonary
Ocular pain (HP:0200026)	1194	1.0	eye
Orthostatic hypotension (HP:0001278)	1478	1.0	neurological
Parageusia (HP:0031249)	638	1.0	neurological
Parkinsonism (HP:0001300)	< 20	1.0	neurological
Petechiae (HP:0000967)	< 20	1.0	skin
Pharyngalgia (HP:0033050)	1782	1.0	ENT
Photophobia (HP:0000613)	39	1.0	eye
Pleural thickening (HP:0031944)	< 20	1.0	pulmonary
Postexertional malaise (HP:0030973)	< 20	1.0	constitutional
Pruritus (HP:0000989)	486	1.0	skin
Pulsatile tinnitus (HP:0008629)	256	1.0	ENT
Recurrent fever (HP:0001954)	111	1.0	endocrine

Continued on next page

Table S3 – continued from previous page

HPO Term	count	p-val (adj)	category
Red eye (HP:0025337)	< 20	1.0	eye
Renal insufficiency (HP:0000083)	446	1.0	endocrine
Restrictive ventilatory defect (HP:0002091)	22	1.0	pulmonary
Rhinitis (HP:0012384)	46	1.0	ENT
Rhinorrhea (HP:0031417)	108	1.0	pulmonary
Scaling skin (HP:0040189)	< 20	1.0	skin
Skeletal muscle atrophy (HP:0003202)	468	1.0	neurological
Skin rash (HP:0000988)	10708	1.0	skin
Sleep disturbance (HP:0002360)	269	1.0	neurological
Sneeze (HP:0025095)	264	1.0	pulmonary
Somatic sensory dysfunction (HP:0003474)	22	1.0	neurological
Spasticity (HP:0001257)	27	1.0	neurological
Stroke (HP:0001297)	153	1.0	cardiovascular
Urinary incontinence (HP:0000020)	2422	1.0	endocrine
Venous thrombosis (HP:0004936)	87	1.0	cardiovascular
Vitreous floaters (HP:0100832)	1281	1.0	eye
Vomiting (HP:0002013)	6353	1.0	gastrointestinal
Wheezing (HP:0030828)	4832	1.0	pulmonary
Xerostomia (HP:0000217)	485	1.0	constitutional
